# Extending the IMQ Model: Deep Characterization of the Human TLR7 Response for Early Drug Development

**DOI:** 10.1007/s10753-024-02127-x

**Published:** 2024-08-26

**Authors:** Juliette A. van den Noort, Salma Assil, Micha N. Ronner, Michelle Osse, Iris Pot, Yalçin Yavuz, Jeffrey Damman, Erik Lubberts, Robert Rissmann, Tessa Niemeyer-van der Kolk, Ingrid Tomljanovic, Manon A. A. Jansen, Matthijs Moerland

**Affiliations:** 1 Centre for Human Drug Research, Zernikedreef 8, 2333CL Leiden, The Netherlands; 2 Leiden University Medical Centre, Leiden, The Netherlands; 3 Erasmus University Medical Centre, Rotterdam, The Netherlands; 4 Leiden Academic Centre for Drug Research, Leiden University, Leiden, The Netherlands; 5 Department of Dermatology, Leiden University Medical Centre, Leiden, The Netherlands

**Keywords:** TLR7, Imiquimod, Prolonged exposure, Challenge

## Abstract

Imiquimod (IMQ; brand name Aldara®) is a registered topical agent that has been proven to induce local inflammation via the Toll-like receptor (TLR)7 pathway. The purpose of this study was to characterize TLR7-mediated inflammation following 7 days (168 h) of topical IMQ exposure in healthy volunteers, and to compare the effects of short exposure (48 h-72 h) with prolonged exposure (120 h-168 h). IMQ (100mg) was applied under occlusion to 5 different tape-stripped treatment sites on the back of 10 healthy participants for a maximum of 7 consecutive days. Erythema and skin perfusion were measured daily up to 168h. Biopsies for immunohistochemical staining and RNA sequencing were collected at 0h, 48h, 72h, 120h and 168h post IMQ application. IMQ triggered an inflammatory response starting at 48h after application, including erythema and perfusion of the skin. At the transcriptomic level, IMQ induced TLR7 signalling, IRF involvement and activation of TNF signalling via NF-κB. Furthermore, an enhanced inflammatory response at the cellular level was observed after prolonged IMQ exposure, with cellular infiltration of dendritic cells, macrophages and T cells which was also corroborated by transcriptomic profiles. No difference was found in the erythema and perfusion response after 168h of IMQ exposure compared to 72h. Prolonged IMQ exposure revealed enhanced cellular responses and additional pathways with modulated activity compared to short exposure and can therefore be of interest as a model for investigational compounds targeting innate and adaptive immune responses.

**Supplementary Information:**

The online version contains supplementary material available at 10.1007/s10753-024-02127-x.

## Introduction

Mouse models are the backbone of the preclinical development of investigational compounds, but in the field of inflammatory diseases, species differences can be pronounced and can hamper the translational step from animals to humans [1, 2]. In this context, human innate immune challenge models are a valuable tool to mimic components of the pathophysiology of a disease state in a healthy individual. Innate immune challenge models can thereby unravel underlying human physiological processes and facilitate the evaluation of pharmacological effects of investigational compounds in early clinical development.

Imiquimod (IMQ) is primarily an agonist of the Toll-like receptor (TLR)7, an endosomal TLR which recognizes single stranded RNA from viruses. IMQ is marketed as a 5% cream (Aldara®) for the treatment of (pre)malignant and HPV-induced skin lesions because of its antiviral and tumoricidal effects [3]. The antiviral and tumoricidal effects are attributed to the attraction of TLR7-bearing monocytes, macrophages and plasmacytoid dendritic cells (pDCs), which subsequently produce proinflammatory cytokines and chemokines and attract other immune cells to the application site [4]. Topical IMQ has been used as a challenge agent to induce psoriasis-like skin inflammation in mice [4–6]. In these studies, mice were exposed to IMQ for 120h to 168h, resulting in a dose-dependent clinical inflammation (i.e. increased ear thickness, erythema and scaling) for the entire duration of the treatment [4, 5]. The clinical inflammation was accompanied by a substantial influx of T cells, conventional dendritic cells (DCs) and pDCs, with an essential role for the interleukin (IL) 23/IL-17 axis [4, 6]. Additionally, research showed that the murine IMQ response was driven by neutrophil influx and complement factor C3 [5].

IMQ was previously used as a human pharmacological challenge agent in multiple studies, where it was topically applied for 48h or 72h [7, 8]. In these studies, IMQ application resulted in a transient, mild to moderate local skin inflammation with a significant increase in skin erythema and perfusion peaking 48h after the first application. Consistent with the working mechanism of IMQ, the response was accompanied by a clearly increased expression of Mx-A, an interferon-driven protein, suggesting engagement of Interferon Regulatory Factor (IRF)7 signalling [7, 8]. Interestingly, the cellular and molecular responses after 48h-72h were relatively mild with a moderate influx of monocytes, natural killer (NK) cells and DCs, mild IL-6 production, and no significant deposition of complement. There was almost no involvement of neutrophils in this model after 48h-72h of IMQ application [8]. The lack of neutrophil involvement is surprising given the molecular signalling of TLR7 [4], and contrasts the preclinical findings in mice, in which neutrophils play a more prominent role. Notably, the duration of the exposure to IMQ in clinical studies has never exceeded 72h, whilst preclinical studies usually span up to 6 days. Characterization of IMQ-induced skin inflammation in healthy participants following extended exposure may therefore elucidate valuable novel aspects of the model and the underlying human immune response.

The purpose of this study was to characterize TLR7-mediated inflammation following 7 days (168h) of IMQ exposure in healthy volunteers. We aimed to provide deeper insights into the translational value of the IMQ model for future early-stage clinical studies, particularly for the investigation of the pharmacological activity of innate immune-targeting compounds.

## Materials and Methods

This clinical study was a single-centre, open-label, investigator-initiated inflammatory challenge study executed in accordance with the Dutch Act on Medical Research involving Human Subjects (WMO). The study protocol was approved by a Medical Ethics Committee (Stichting Beoordeling Ethiek Biomedisch Onderzoek, Assen, the Netherlands). Written informed consent was obtained from all subjects prior to any study-related procedures.

## Study Design and Inclusion

We recruited ten healthy male and female volunteers between the ages of 18 and 45 and with Fitzpatrick skin types I-III. Their health status was assessed by means of medical history, physical examination, laboratory tests, and 12-lead electrocardiograms (ECG). Participants were excluded if they had a familial history of psoriasis, pathological skin conditions in the treatment area, prior experience with hypertrophic scarring or keloid, or if they were exposed to IMQ within three months of enrolment.

## Treatment

**Fig. 1:**
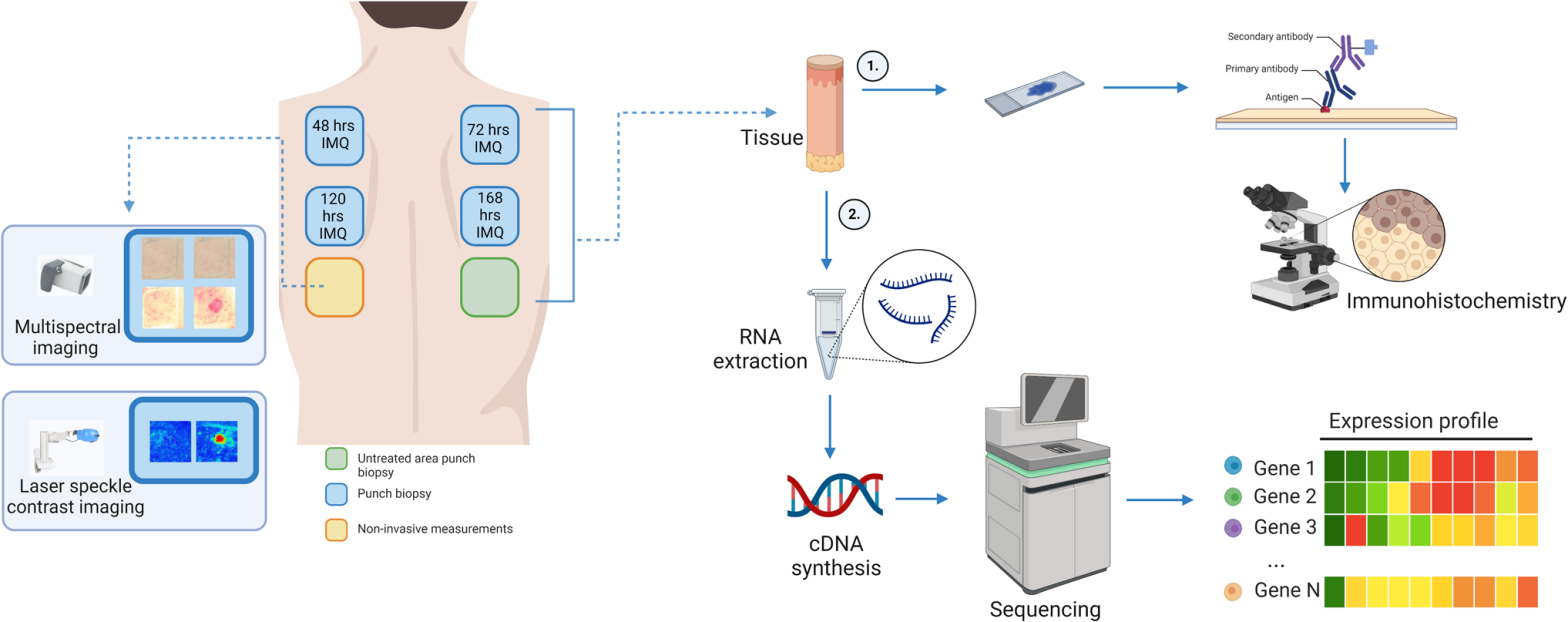
Overview of the study design. IMQ was topically applied to five treatment sites on the back of healthy participants for a maximum of seven consecutive days (168h). At specified time intervals (48h, 72h, 120h and 168h), 4-mm biopsy samples were obtained from the IMQ-treated regions as well as from the untreated region, resulting in a total of 5 biopsies per volunteer. Endpoints included non-invasive imaging, immunohistochemical staining and RNA sequencing of biopsy material. Abbreviations: cDNA = complement DNA, hrs = hours, IMQ = imiquimod. *Created with BioRender.com*.

IMQ was topically applied to five treatment sites on the back for a maximum of seven consecutive days (168h). The back was marked with six squares: one untreated area and five treatment areas (Fig. 1). Each treatment area was tape stripped (D-Squame, CuDerm, Dallas, TX) to induce mild skin barrier disruption until a trans-epidermal water loss (AquaFlux, Biox Systems) value of 20g/m^2*^h was reached. After tape stripping, a standard daily dose of 5 mg IMQ (100mg Aldara® 5%) was applied under occlusion using a 12mm Finn chamber (Bipharma, Almere, the Netherlands), which was renewed once every 24h. In this article, we define “short exposure” as the application of IMQ for 48h-72h, and “long exposure” as IMQ application in the duration of 120h-168h.

## Skin Assessments

To evaluate the inflammatory skin response, subjects underwent sequential assessments prior to IMQ challenge (0h) and at 48h, 72h, 120h and 168h post IMQ challenge, as well as during follow up (14 days after first IMQ application). One treatment site was selected to evaluate endpoints of non-invasive procedures only, throughout the duration of the study (Fig. 1). Erythema was assessed by a physician using a 4-point scale ranging from 0 (absent) to 3 (severe). Additionally, erythema and skin perfusion were assessed by means of multispectral imaging analysis (Antera 3D, Miravex, Ireland) and laser speckle contrast imaging (LSCI, PeriCam PSI System, Perimed Jäfälla, Sweden), respectively. All skin assessments were performed under standardized conditions with a room temperature between 20–24 degrees Celsius (°C).

## Biopsy Processing

At specified time intervals (Fig. 1), 4-mm biopsy samples were obtained from the IMQ-treated regions as well as from the untreated region, resulting in a total of 5 biopsies per volunteer. One part of the biopsy was fixed in 4% formaldehyde at 4°C for 24-48h and subsequently transferred to 70% ethanol at room temperature. The other part was rapidly frozen in gelatine capsules containing Tissue Tek OCT medium (Sakura Finetek USA, Inc., Torrance, USA) and stored in liquid nitrogen until immunohistochemistry (IHC) staining at the pathology Laboratory of Erasmus Medical Centre, Rotterdam, the Netherlands.

## IHC and Direct Immunofluorescence (DIF)

IHC staining was performed for the following targets: CD11c (Clone EP157, Bio SB), CD14 (Clone EPR3653, Ventana), CD20 (Clone L26, Ventana), CD1a (Clone EP3622, Cell Marque), CD3 (Clone 2GV6, Ventana), CD4 (Clone SP35, Ventana), CD8 (Clone C8/144B, DAKO), HLA-DR (Clone CR3142, Ventana), MPO (Polyclonal, Ventana), and NF-κB (Clone d14e12, Cell Signaling). Biopsies were scored by a clinical pathologist using a 6-point nominal scale: negative (0), minimal (1), few (2), moderate (3), many (4), excessive (5). DIF was applied for complement C3c (rabbit polyclonal, DAKO, Glostrup, Denmark) and C4d (rabbit polyclonal, Biomedica, Wien, Austria). DIF intensity was scored by a clinical pathologist on a nominal scale of 0–3: none (0), weak (1), moderate (2) and strong (3).

## RNA Isolation, Sequencing, and Data Preprocessing

The remaining snap frozen tissue was lysed using RLT lysis buffer with β‐mercaptoethanol and extracted using the RNeasy micro plus kit (Qiagen, cat no. 74034). The extracted RNA concentration was assessed using the Molecular Probes Quant iT RNA HS Assay Kit (ThermoFisher Scientific, cat no. Q32852). A set of 45 samples yielded sufficient amounts of RNA for sequencing at Genomescan BV, Leiden. RNA libraries were constructed using the NEBNext Ultra II Directional RNA Library Prep Kit from Illumina (New England BioLabs, Ipswich, MA, USA, cat. no. E7760S/L). Samples were prepared using the NEBNext® Poly(A) mRNA Magnetic Isolation Module. mRNA was isolated using oligo-dT magnetic beads, followed by RNA fragmentation, cDNA synthesis, adapter ligation and PCR amplification of the cDNA library. Bulk RNA sequencing was performed to obtain 40 million reads per sample using the Illumina NovaSeq 6000, yielding 150 bp paired end reads. Raw sequencing reads were processed as follows: adapter trimming and filtering of low-quality bases using fastpv0.23.2, alignment to GRCh38.p13 human reference using STAR2 v2.7.10 and gene level raw count quantification using HTSeq version 2.0.2.

## RNA Sequencing Data Analyses

Data visualization and statistical analyses of the RNA sequencing data were performed using R statistical software (v4.3.1) [9]. An overview of samples and associated information is provided in Supplemental Table S1. t-distributed Stochastic Neighbour Embedding (t-SNE) was used as an unsupervised dimensionality reduction approach to visualize the intrinsic structure of the dataset. The algorithm was applied to *DESeq2* variance-stabilized (vst) counts of 2000 most variably expressed genes using the *Rtsne* package (v0.16) [10–12]. Based on transcriptomic profiles, a set of 15 IMQ-treated samples co-clustering with the untreated samples were designated as putative (partial or) non-responders. The molecular response to IMQ was investigated in the transcriptomes of responders, using differential gene expression and subsequent pathway analyses. Differential gene expression analysis was performed using the *DESeq2* package (v1.40.2) with subsequent LFC shrinkage using the ‘apeglm’ estimator [13, 14]. Gene set enrichment analysis (GSEA) was performed on a curated subset of the Molecular Signatures Database (MSigDB) v2023.2.Hs using the ​*clusterProfiler* package (v4.8.2) [15–18]. Databases referenced included: hallmark gene sets, oncogenic signatures Gene Ontology, 3CA, miRNA and transcription factor targets, curated gene sets from Wikipathways, PID, Reactome, Biocarta and KEGG/KEGG Medicus. For the MSigDB hallmark gene set collection [18], pathway activity was additionally assessed at the single-sample level using Gene Set Variation Analysis (GSVA) applied with the *GSVA* package (v1.48.3) [19]. Hierarchically clustered gene expression heatmaps were generated using the *pheatmap* package (v1.0.12) [20]. Plots linking core enrichment genes and enriched pathways were drawn using the cnetplot function of the *enrichplot* package (v1.20.3) [21]. Gene expression boxplots were produced using the DESeq2 plotCounts function and further modified with *ggplot2* (v3.5.0). CIBERSORTx [22] was used to quantify the abundances of 22 immune cell type populations across all samples in the RNA sequencing dataset. The LM22 signature matrix was used as the reference profile and B-mode batch correction was applied. The Skillings-Mack test was used to assess the differences in absolute scores for each cell type across five sample groups (untreated, IMQ48h, IMQ72h, IMQ120h, IMQ168). The Conover’s all-pairs test was applied with the PMCMRplus package (v1.9.10) as the post hoc test using data from subjects with complete observations. Unless otherwise specified, the statistical significance threshold across all analyses was set at 0.05 and a Benjamini–Hochberg correction was applied to account for multiple testing.

## Complement Analysis in Blood Samples

Blood was collected in a 4 mL Clot activation Tube (CAT) and plasma in a 4 mL K2EDTA collection tube. Concentrations of the complement components C3, C3d, C3d/C3 ratio and the soluble membrane attack complex C5b-9 were measured in plasma at the laboratory of the Department of Nephrology, University Medical Centre Groningen as described earlier [23].

## Statistics

All repeatedly measured pharmacodynamic (PD) endpoints were summarised (n, mean, standard deviation (SD)) by area and time. Repeatedly measured continuous PD endpoints were analysed using a mixed model analysis of covariance (ANCOVA) with area (48h IMQ, 72h IMQ, 120h IMQ, 168h IMQ and untreated), hours (0h, 48h,72h,120h and 168h) and area by hours as the fixed factors and subject as the random factor and the covariate baseline measurement (when applicable). A summary table of the analysis results per variable was generated with estimates of the differences between the contrasts and a back transformed estimate of the differences in percentage for log transformed parameters, 95% confidence intervals (in percentages for log-transformed parameters) and Least Square Means (geometric means for log transformed parameters) with corresponding p-values.

## Results

### Study Population and Adverse Events

Ten healthy volunteers were enrolled, 7 of whom were female and 3 were male. General subject characteristics are provided in Supplemental Table S2. All subjects had Fitzpatrick skin type I-III and a mean age of 25.6 (SD ± 6.7) years. The most frequently reported adverse event was application site pruritus, which was generally transient and disappeared spontaneously after IMQ application was stopped. No serious adverse events were reported.

## IMQ Treatment Drives Erythema and Skin Perfusion, but Long Exposure Does not Enhance these Responses

**Fig. 2:**
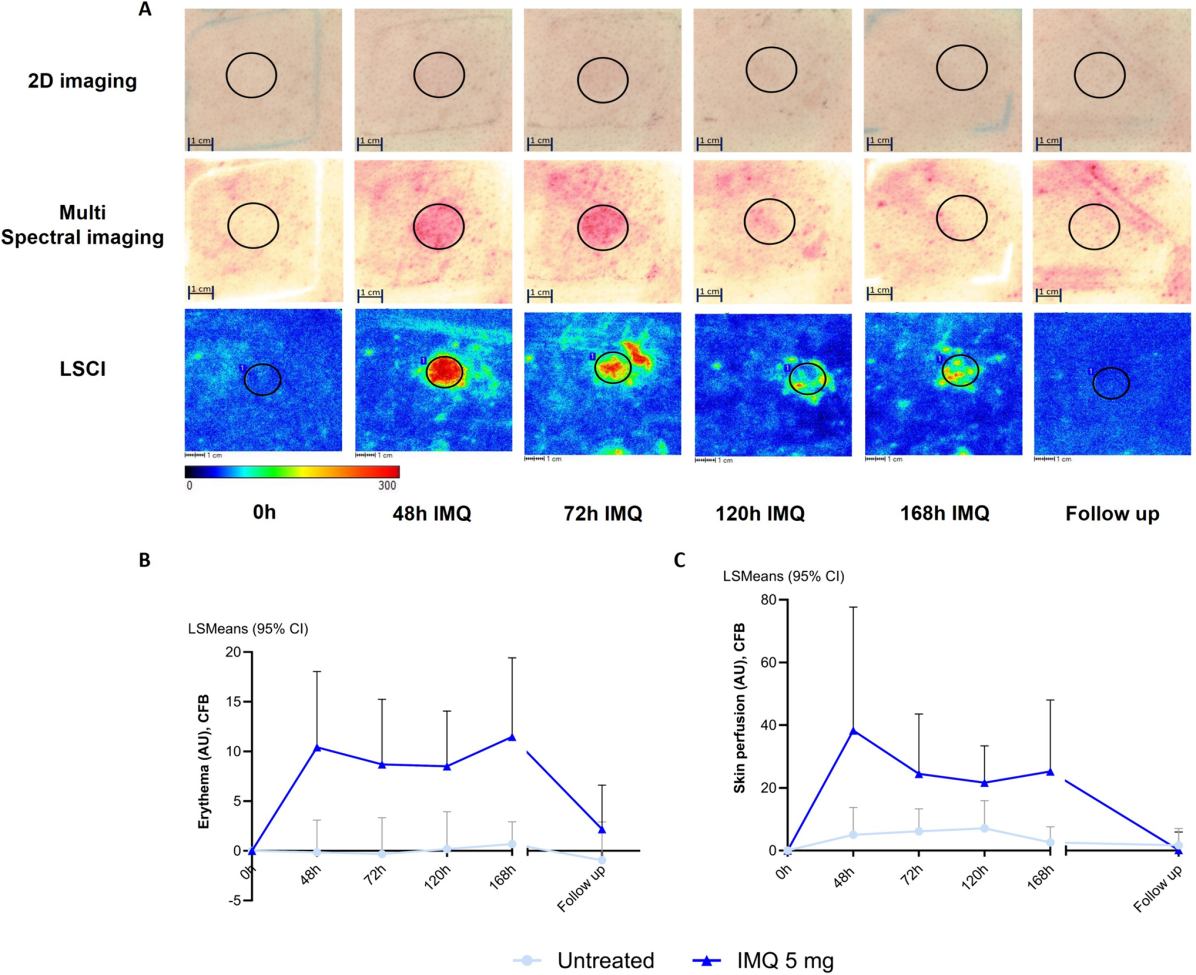
Clinical impression and quantification of inflammatory skin response by multispectral imaging and LSCI. **A** Overview of 2D imaging, LSCI and multispectral imaging. **B** Erythema measured by multispectral camera, illustrated as CFB (n = 10 for 0-168h, n = 9 for follow up, imaging data of 1 subject were missing at follow up (D14) as D14 visit took place outside of the allowed time window). No difference observed between short (48h) and long (168h) IMQ exposure, estimated difference: 1.07, 95% CI [-3.67, 5.80], p = 0.6567. **C** Skin perfusion by LSCI, illustrated as CFB (n = 10 for 0-168h, n = 9 for follow up). No difference observed between short (48h) and long (168h) IMQ exposure, estimated difference: -12.8%, 95% CI [-29.7%, 8.1%], p = 0.2093. Abbreviations: CFB = change from baseline, CI = confidence interval, IMQ = imiquimod, LSCI = laser speckle contrast imaging, LSMeans = Least Squares Mean.

A visual representation of all imaging and biophysical assessments is provided in Fig. 2A. IMQ application under occlusion for 168h led to a significant increase in erythema (estimated difference (ED): 7.69, 95% confidence interval (CI) [5.51, 9.86], p < 0.0001) and blood perfusion (ED: 25.1%, 95% CI[13.5%, 37.9%], p < 0.0001) compared to untreated (Fig. 2A-C). Comparison of the response after 168h of IMQ versus 0h revealed a similar significant difference for erythema (11.48, 95% CI[6.75, 16.21], p < 0.0001) and for perfusion (ED: 41.6%, 95% CI[14.2%, 75.6%], p < 0.0018). The erythema and perfusion response peaked at 48h with subsequent decline over time. No significant differences in erythema (ED: 1.07, 95% CI[-3.67, 5.80], p = 0.6567) or perfusion (estimated difference: -12.8%, 95% CI[-29.7%, 8.1%], p = 0.2093) were observed upon long IMQ exposure (168h) compared to short exposure (48h; Fig. 2B-C).

## IMQ Triggers an Inflammatory Response at the Transcriptomic Level

**Fig. 3:**
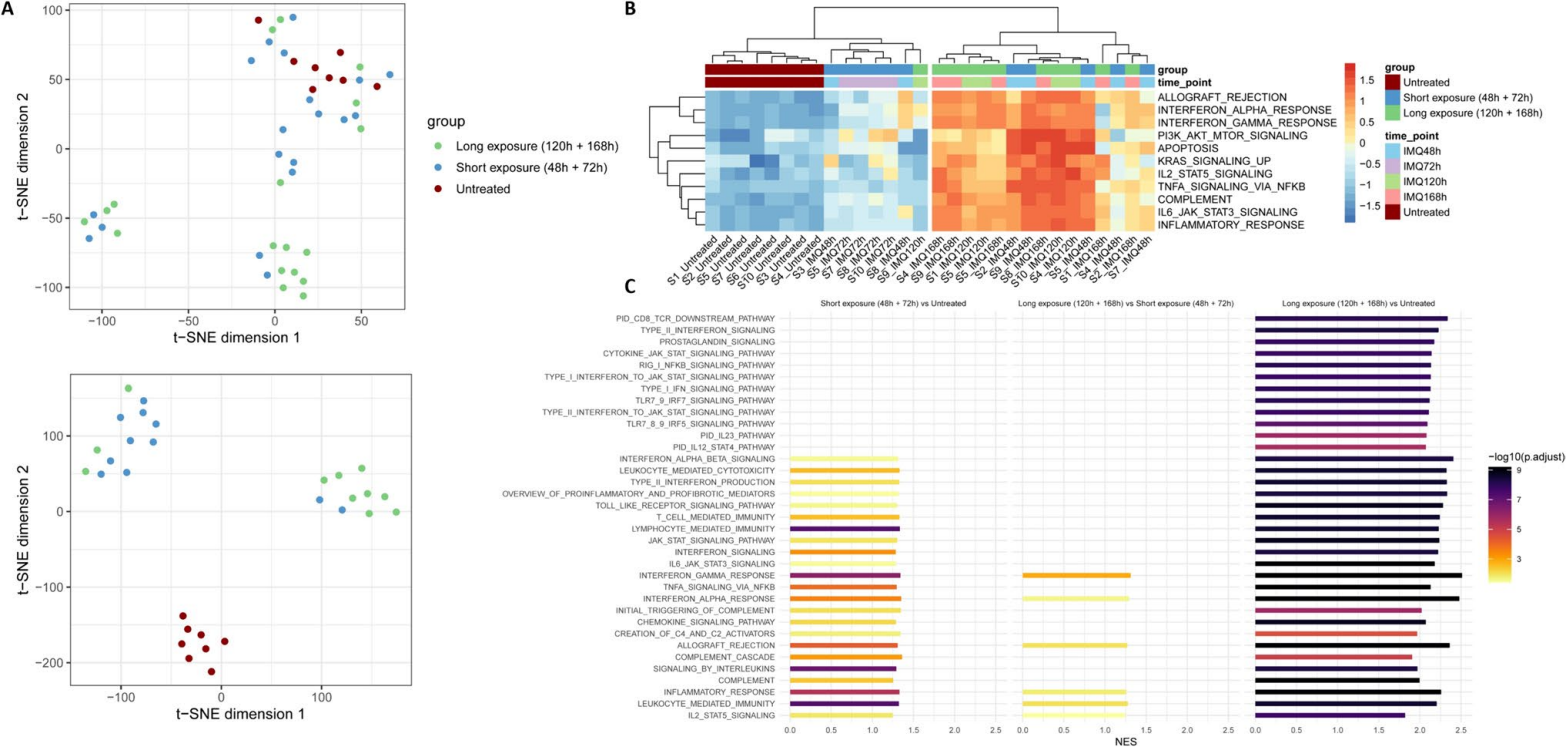
Transcriptomic profiling of imiquimod response in tissue biopsies **A** t-SNE plot visualizing the clusters present in the full dataset (n = 45, top) and a reduced subset of the dataset (n = 30, bottom) excluding the treated samples which showed similarity to the untreated group (putative non-responders based on their transcriptomic profiles). **B** Heatmap of GSVA single-sample pathway enrichment scores on 11 representative MSigDB Hallmark gene sets (n = 30). IMQ application leads to activation of pathways involved in inflammatory and immune responses, including TNF signalling via NF-κB, IFN-α and IFN-γ responses, and complement pathways. **C** Barplots displaying GSEA normalized enrichment scores of 35 representative pathways upregulated upon IMQ exposure for the following comparisons: short exposure versus untreated (left), long exposure versus short exposure (middle) and long exposure versus untreated (right), based on the reduced dataset (n = 30). Abbreviations: GSEA = gene set enrichment analysis, GSVA = gene set variation analysis, IFN = interferon, IMQ = imiquimod, NES = normalized enrichment score, NF-κB = nuclear factor kappa-light-chain-enhancer of activated B cells, TNF = tumour necrosis factor, t-SNE = t-distributed Stochastic Neighbour Embedding.

Analysis of the RNA sequencing dataset using t-SNE revealed three major clusters predominantly enriched in samples from the untreated group, short exposure time points (48h-72h) and long exposure time points (120h-168h) (Fig. 3A). Based on transcriptomic profiles, we grouped samples from the short exposure time points (48h-72h) and samples from the long exposure time points (120h-168h) for downstream analysis. The short exposure group (48h-72h) was compared to the long exposure group (120h-168h), and both groups were also compared to the untreated group. Pathway analysis of hallmark gene sets demonstrated that IMQ application activates the following inflammatory pathways: TNF signalling via NF-κB, IFN-α and IFN-γ responses, and complement pathways, which were most prominently activated after long exposure to IMQ (Fig. 3B, Supplemental Fig.S1).

## IMQ Exposure Induces TLR7 Signalling and Activation of TNF Signalling via NF-κB

**Fig. 4:**
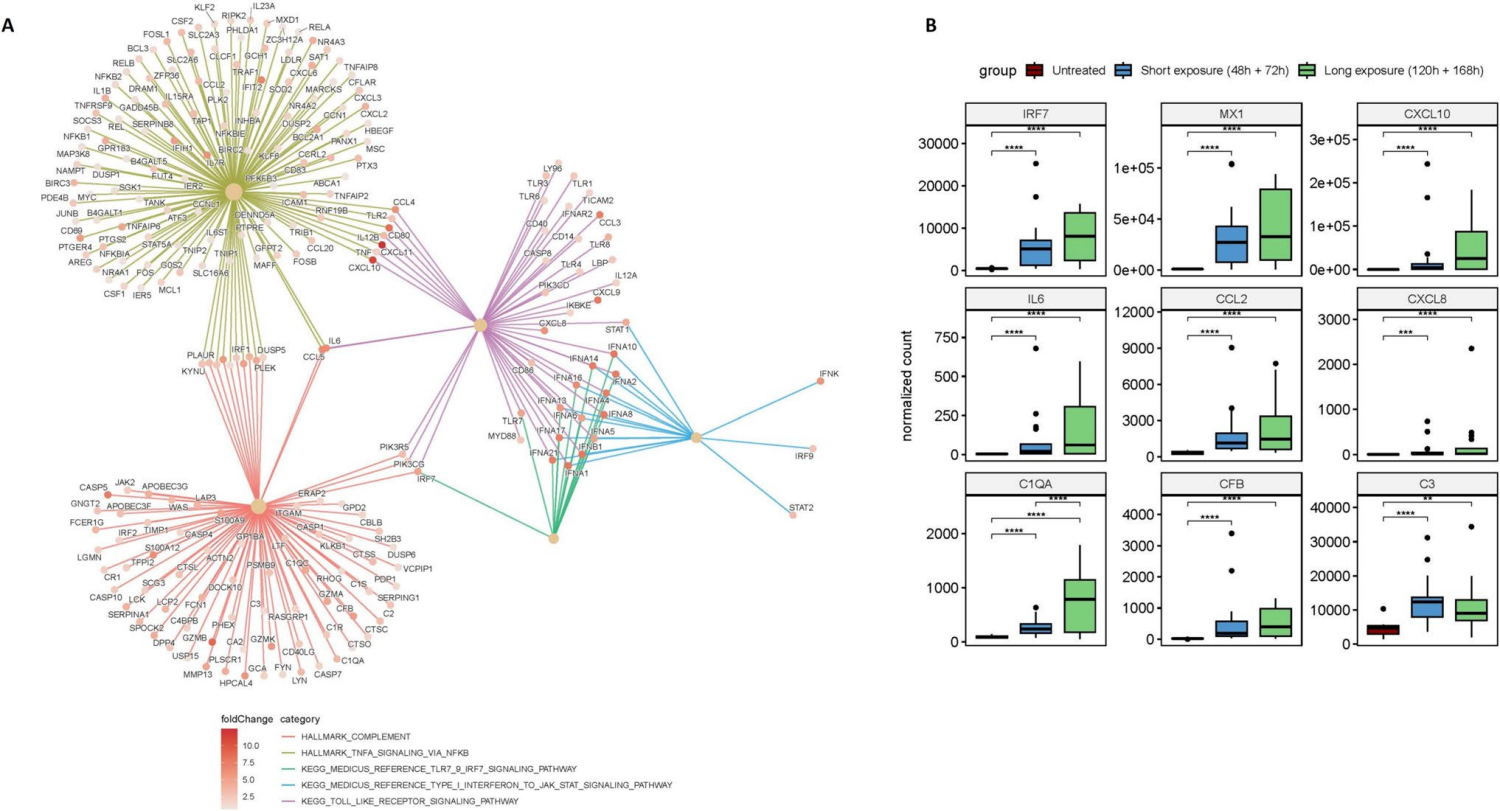
Key pathways activated after long imiquimod exposure **A** Network plot displaying selected biological pathways involved in the IMQ response and their associated core enrichments genes. Colours of network edges correspond to each biological pathway including complement, TNF-signalling via NF-κB, TLR7/9/IRF7 Signalling, Type I interferon to JAK-STAT signalling and TLR signalling. Colours of nodes correspond to the log2 fold change values for individual genes based on the late exposure versus untreated group comparison. **B** Boxplots of DESeq2 normalized counts with an added pseudocount of 0.5 shown for 9 selected DEGs involved in the TLR-induced IRF7 signalling (top row), NF-κB signalling (middle row) and complement activation pathways (bottom row). Statistical significance corresponds to adjusted p-values from DESeq2 differential expression analysis for each given comparison.

We further investigated the biological processes involved in the different stages of exposure to IMQ using GSEA (Fig. 3C). Short IMQ exposure led to activation of TLR signalling pathways, interferon-driven responses and TNF signalling via NF-κB (Fig. 3C). Upon longer IMQ exposure, activation of TLR-induced IRF7 signalling was revealed and TNF signalling via NF-κB became more prominent (Fig. 3C). Additionally, downstream effects representative of the TLR pathway including induction of type I and type II interferons leading to the activation of the JAK-STAT pathway were identified after long exposure (Fig. 3C). When comparing long to short IMQ exposure, a positive enrichment of gene sets relating to IFN-α and -γ was found, as well as additional activated pathways including IL-2 signalling (Fig. 3C). A network plot was generated displaying the linkages between the key biological pathways involved in the prolonged IMQ response (Fig. 4A). We next focused on a selection of relevant differentially expressed genes (DEGs) involved in TLR-induced IRF7 signalling, NF-κB signalling and complement activation pathways (Fig. 4B; full pathways shown in Supplemental Fig.S2). For IRF signalling, a time-dependent increase was found in the expression of genes encoding for IRF7, Mx-1 and CXCL10 in IMQ-treated samples compared to untreated samples (Fig. 4B). RNA sequencing-based NF-κB responses were confirmed by immunohistochemical staining of skin punch biopsies: NF-κB (total) staining was elevated between 120 and 168h post IMQ application, compared to baseline (Supplemental Fig. S3C). Downstream of NF-κB, we found a similar increase in expression for IL-6 and CCL2 (Fig. 4B). CXCL8 expression was upregulated after IMQ application, but the difference in expression was not significant between long and short exposure. An overview of expression for markers corresponding to the IHC staining is available in Supplemental Fig. S4.

## IMQ-Driven Expression of Complement Genes

Transcriptomic analysis at the pathway level showed a positive enrichment of gene sets related to the complement cascade, with stronger enrichment upon prolonged exposure (Fig. 3C). Complement genes elevated in expression by IMQ application included C1QA, C3 and CFB (Fig. 4B). Results for the transcripts encoding for complement proteins downstream of C3 were inconsistent, with mixed expression levels between samples (Supplemental Fig. S2B). IHC staining for complement revealed traces of dermal C4d at baseline, but no deposition of C3c or C4d after 168h of exposure to IMQ. Complement proteins showed no systemic elevation of C3, C3d, C3d/C3 or C5b-9 in plasma after 168h of IMQ-exposure (not shown).

## Increased Cellular Infiltration After long IMQ Exposure

**Fig. 5:**
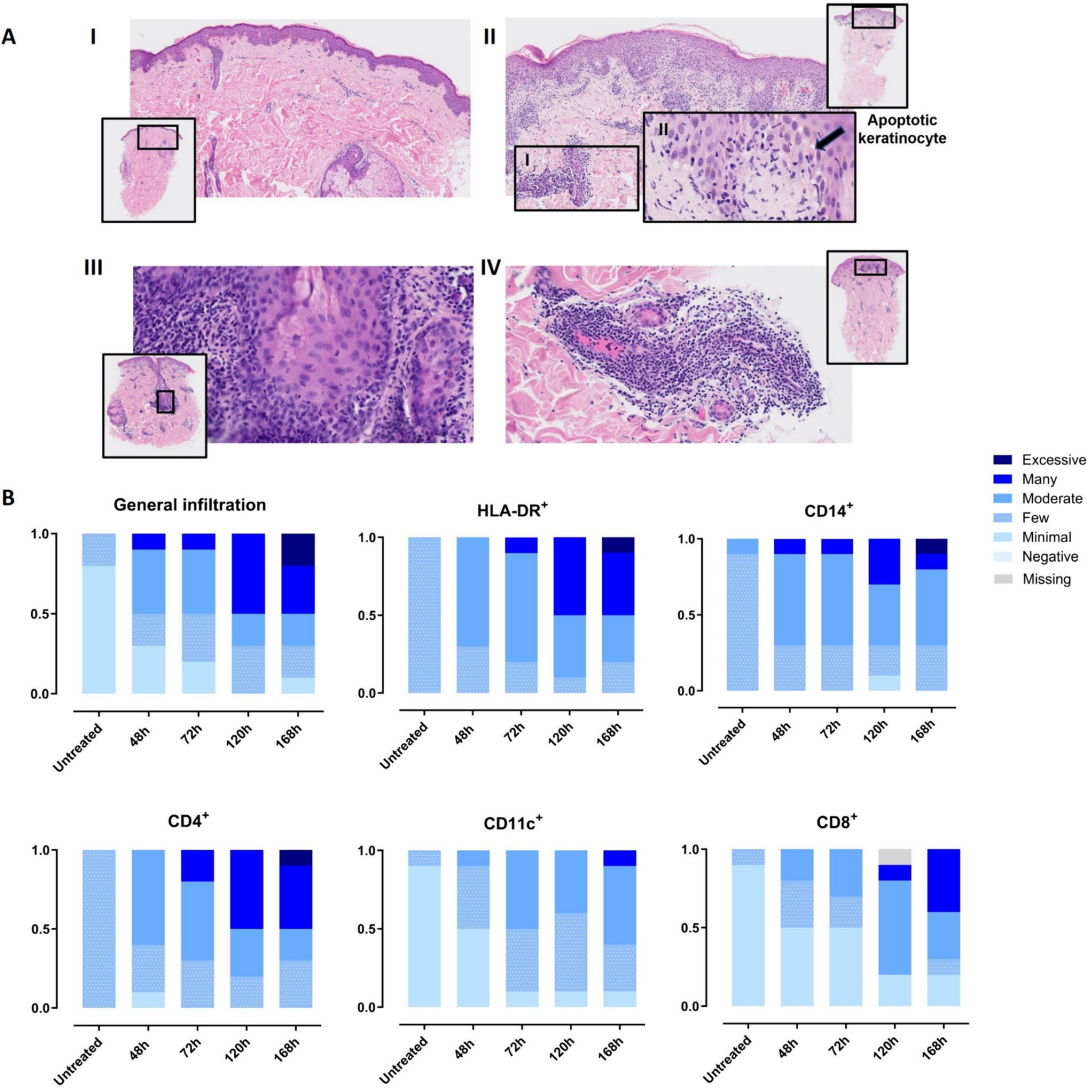
Enhanced inflammatory responses at histological and cellular levels observed upon prolonged IMQ exposure. **A** histological sections. Section *I)* Untreated skin, shows healthy skin without any signs of inflammation (zoom factor 5). *II)* Section of 72 h post IMQ application showing an abundant lymphocytic exocytosis in the epidermis and superficial lymphohistiocytic perivascular infiltrate (zoom factor 5). Inset I shows exocytosis of lymphocytes in a sweat gland (zoom factor 5). Inset II shows lymphocytic exocytosis in the epidermis accompanied by an apoptotic/necrotic keratinocyte (arrow, zoom factor 40). *III)* Section of 120 h of IMQ application showing profound perifollicular inflammation and influx of lymphocytes in the hair follicle epithelium (zoom factor 20). *IV)* Section of 168 h of IMQ application resulting in cuffing/lymphocytic vasculopathy of the infiltrate surrounding the deep vascular plexus at the levels of the sweat glands (zoom factor 20). **B** General infiltration and IMQ-driven immune cells (macrophages, monocytes, T helper cells, dendritic cells and cytotoxic T cells) measured by IHC in skin punch biopsies. Abbreviations: IHC = immunohistochemistry, IMQ = imiquimod.

Histologically, the general inflammation pattern after IMQ exposure was a lymphohistiocytic perivascular dermatitis with an increasing degree and deeper extension of inflammation over time (Fig. 5A). In 9/10 individuals the infiltrate showed peri-adnexal (peri-follicular and/or peri-eccrine) involvement and epidermal interface dermatitis (6/10). A rise in general cell infiltration accompanied by a mild increase in acanthosis as well as lymphocytic exocytosis (Supplemental Fig. S3A,B) was evident upon prolonged IMQ exposure (Fig. 5B). Immunohistochemical staining showed infiltration of monocytes, DCs and macrophages (Fig. 5B) but no presence of neutrophils (Supplemental Fig. S3E). The cells were present after 48h-72h, with infiltration more elevated after 120h-168h post IMQ application. This pattern was mirrored by the T cell response, which showed a rise in T helper cells and cytotoxic T cells until 168h. (Fig. 5B). A minimal number of Langerhans cells and B cells was present (Supplemental Fig. S3D,F).

**Fig. 6:**
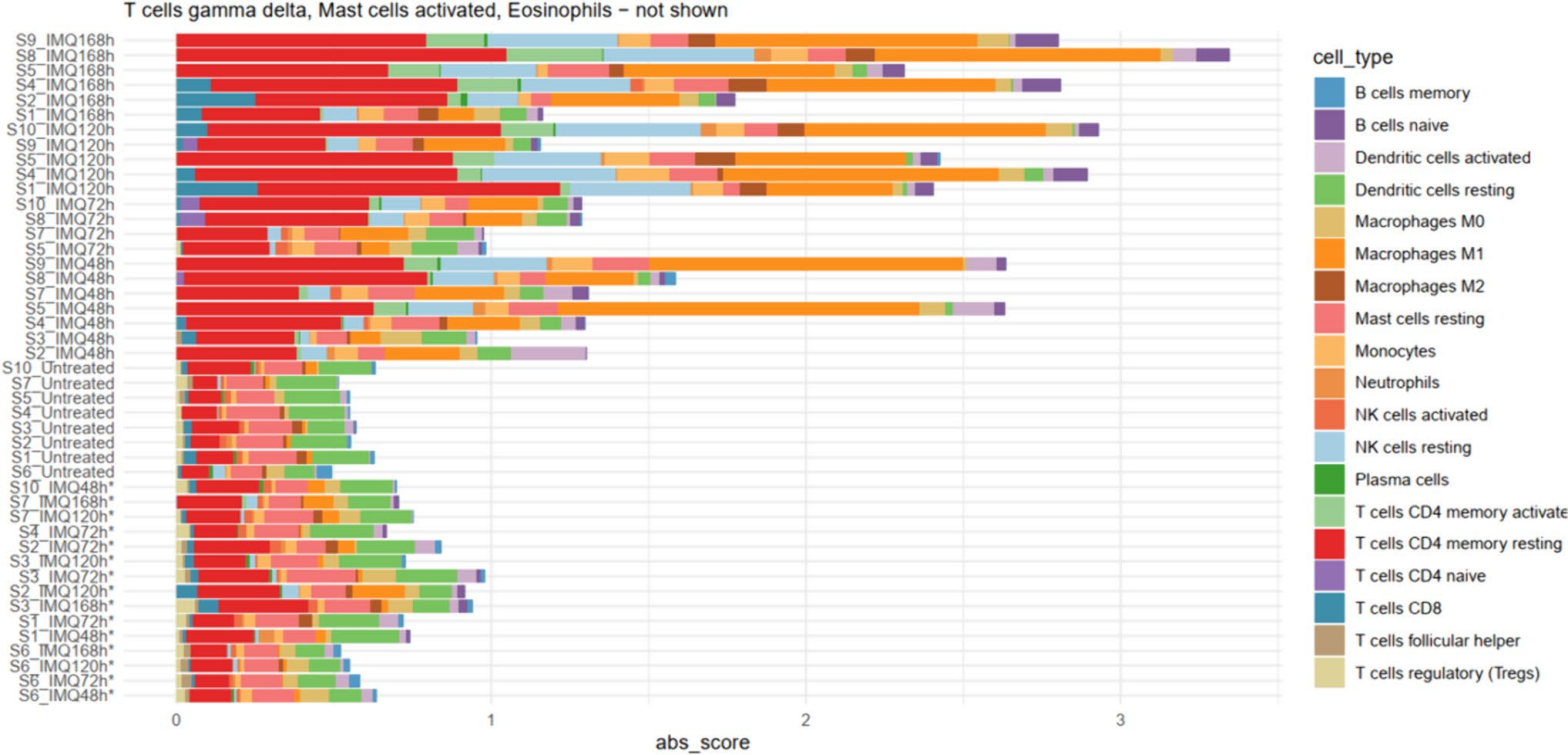
Overview of immune cell type infiltration based on CIBERSORTx. Stacked barplot displaying the absolute scores for 19 cell types detected by CIBERSORTx across all samples (n = 45). An asterisk (*) refers to putative molecular non-responders based on transcriptomic profile similarity to untreated samples. Abbreviations: IMQ = imiquimod, NK = natural killer

The CIBERSORTx algorithm identified a total of 19 out of 22 immmune cell subpopulations (Fig. 6). IMQ application generally led to an increase in immune cell type absolute scores compared to the untreated samples. A globally significant difference (using Skillings-Mack test) between groups is observed, among others, for resting NK cells (p = 0.0292), naïve B cells, (p = 0.0051), M1 Macrophages (p = 0.0003), M2 macrophages (p = 0.0403), activated DCs (p = 0.001), resting CD4^+^ memory T cells (p = 0.0003) and activated CD4^+^ memory T cells (p = 0.0241), and CD8^+^ T cells (p = 0.0463) depicted in Supplemental Fig. S7. Of these, naïve B cells, M1 macrophages, resting NK cells and both resting and activated CD4^+^ memory T cells showed significant increase in abundance at 168h vs. 72h after IMQ exposure in the post-hoc analysis. Additionally, a statistically significant reduced abundance was observed in both resting and activated DCs at 168h vs. 72h. M2 macrophages and CD8^+^ T cells did not show statistically significant changes at individual timepoints. Remarkably, overall low abundance of neutrophils was observed (Supplemental Fig. S5).

## Discussion

This study is the first to elucidate the acute effects of long-term IMQ exposure using a multimodal approach in healthy volunteers. We have shown that compared to short exposure, long exposure to IMQ results in a stronger immunological response as evidenced by additional enriched pathways such as TLR-induced IRF7 signalling, more prominent TNF signalling via NF-κB along with downstream effects such as induction of type I and type II interferons leading to activation of the JAK-STAT pathway. Furthermore, increased complement gene expression was identified upon long exposure to IMQ [7, 22]. Although imaging and biophysical measurements showed no significantly enhanced response after long IMQ application compared to short exposure, a strong cellular infiltration boost was observed. At the transcriptomic level, this was demonstrated by an increased abundance of M1 macrophages, resting NK cells, and resting and activated CD4 + memory T cells. While a statistically significant increase was observed in M2 macrophages and CD8^+^ T cells following IMQ application, no difference was observed between long and short exposure. The increased abundance of naïve B cells and the appearance of T cells is indicative of both innate and adaptive immune responses involvement. The transcriptomic profile partially aligns with the IHC-based cellular infiltration of macrophages, NK cells and CD4^+^ T cells, demonstrating clear time-dependent effects with increased infiltration after long IMQ exposure. Additionally, IMQ increased the expression of type II interferon-related genes, which aligns with the IHC observation of CD8^+^ cell influx. These cellular findings are consistent with classical TLR signalling. Activation of TLRs is also known to trigger MyD88, IRAK1 and IRAK4, leading to IRF7 and NF-κB signalling, which is in line with our findings. These pathways result in upregulation of transcription factors for several cytokines including type I interferons, TNF, IL-2, IL-6, IL-8, IL-12, IFN-α and chemokines such as macrophage inflammatory protein (MIP)-1α, MIP-1β and monocyte chemotactic protein-1 [24].

Another challenge agent that we use to effectively induce an in vivo TLR response in men is lipopolysaccharide (LPS) [25–28]. Intradermal injection of LPS triggers an acute inflammatory response via TLR4, leading to increased innate immune cell populations including neutrophils, monocytes and dendritic cells. Furthermore, LPS elicits an adaptive immune response, as evidenced by the presence of B and T cells. Elevated levels of IL-6, IL-8, IL-1β and TNF following LPS injection indicate NF-κB involvement. The current study showed moderate activation of NF-κB signalling after long IMQ application, supported by upregulated expression of NF-κB1, NF-κB2, IL-6, CXCL8, CCL2, IL-17C and IL-23A at the transcriptomic level. This contradicts previous studies, as no significant IL-6 and IL-8 responses were observed 72 h after IMQ application, suggesting only mild NF-κB involvement. However, the release of Mx-A (a downstream marker indicative of IFN-α activation through IRF7) was evident [7, 8]. Our current data reinforces this finding, as it suggests both My-D88 transcript expression and downstream IRF7 and Mx-1 activation, which increases upon prolonged IMQ application. The activation of interferons leads to the engagement of their respective receptors, which in turn triggers the JAK—STAT pathway culminating in the release of proinflammatory cytokines [29, 30]. The JAK—STAT pathway was more enriched, with chemokines such as CXCL9, CXCL10 and CXCL11 significantly overexpressed after prolonged IMQ exposure. Recently, Chen et al. provided an overview of studies examining DNA and RNA specific profiles in cutaneous lupus erythematosus (CLE) patients, which indicates an upregulation of innate immune response functions including JAK – STAT signalling, TLR signalling, and pattern recognition receptors. Furthermore, there was a notable increase in the expression of type 1 interferons, along with an upregulated expression of chemokines CXCL9, CXCL10, and CXCL11, which are recognized as characteristic indicators for CLE [31–34]. Although a direct comparison of our data with the existing RNA datasets of CLE patients was not conducted, analysis of pathway activity and overexpressed genes detected upon prolonged IMQ application allows us to conclude that our current model aligns more closely with CLE characteristics than with psoriasiform lesions [7]. In addition, the histopathological changes of a vacuolar interface dermatitis with adnexal involvement were also reminiscent of CLE. These observations differ from the prevalent use of the model in preclinical studies, where the murine IMQ model is typically used to investigate psoriasis-like conditions [6].

In contrast to our cellular observations, imaging and biophysical measurements showed no significantly enhanced response after long IMQ application compared to short exposure. This may be because Aldara (besides its role as a TLR7 agonist) may also act as an exogenous mediator by enhancing transient receptor potential vanilloid 1 channel activity on the primary afferent sensory neuron [35]. Activation of this channel leads to the release of bioactive substances such as nitric oxide (NO). NO can then interact with target cells in the surrounding tissue, including vascular smooth muscle cells. The interaction of NO with smooth muscle cells leads to vasodilation, resulting in increased blood perfusion and erythema [6, 35]. Our observations suggest that the vascular response is independent of the inflammatory process. The role of bioactive, vasodilating substances in the IMQ-induced erythema and perfusion response remains to be further elucidated.

Our second objective was to explore the translational value of the IMQ model. In contrast to the mouse data, IHC staining did not show involvement of complement in the human IMQ response. We hypothesize that this may be due to the difference in severity of the hit, as in mice, the entire surface area of the back is challenged, whereas in humans IMQ is applied to a much smaller relative surface area. Therefore, it is currently unknown whether the observed differences are a result of the magnitude of TLR activation or can be contributed to species differences [1, 2]. However, at the transcriptome level, classical, and alternative pathway genes were enriched, particularly after prolonged exposure to IMQ, suggesting complement involvement in the human IMQ response. It is unclear how these sequencing results translate to the protein level, or if the transient nature of complement explains the lack of IMQ-driven complement responses in IHC analysis. The same holds true for the observed lack of neutrophils and CXCL8 expression, which emphasizes that the role of neutrophils in the human IMQ response needs to be elucidated further [6]. The observed differences between preclinical animal models and the human response may partially be explained by species differences in TLR7 expression [36]. For instance, Bhagchandani et al. described that the expression of TLR7 on neutrophils is higher in mouse than in men [36, 37]. Moreover, expression patterns of TLR7 within a given cell type may differ across tissues and across activation status of the cell [38], further complicating the translational interpretation both across and within species. These findings highlight the complementary value of human challenge models in the development of immune-targeting compound development.

In conclusion, our study provides a comprehensive characterization of the cutaneous response to both short and prolonged IMQ exposure in healthy volunteers by using a multimodal approach. We have demonstrated that prolonging the IMQ exposure has added value by enhancing cellular responses and increasing abundance of specific immune cell types along with stronger activation of a diverse set of pathways, particularly those driven by IRF and related to complement. We also argue that prolonged IMQ application results in a CLE-like cutaneous inflammation, both at the transcriptomic level and from a histopathological perspective. Our results suggest that biophysical and vascular responses are not exclusively driven by cutaneous inflammation. The described discrepancies between preclinical and clinical results, most notably the neutrophil response, illustrate the complementary value of human challenge models in the development of compounds targeting the immune system. This in vivo immune challenge model is of value for future early clinical evaluation of topically or systemically applied anti-inflammatory or immunomodulatory compounds, particularly compounds targeting IRF and JAK-STAT signalling.

## Authorship Contributions

All authors contributed to the study conception and design. Material preparation, data collection and analysis were performed by Juliette van den Noort, Salma Assil, Micha Ronner, Michelle Osse, Jeffrey Damman and Ingrid Tomljanovic. The first draft of the manuscript was written by Juliette van den Noort and Salma Assil and all authors commented on previous versions of the manuscript. All authors read and approved the final manuscript.

## Supplementary Information

Below is the link to the electronic supplementary material.

Supplementary file1 (DOCX 2519 KB)

## Data Availability

The datasets generated during and/or analysed during the current study are available from the corresponding author upon reasonable request.

## References

[1] Junhee Seok, H., Shaw Warren, G.C. Alex, et al. 2013. Genomic responses in mouse models poorly mimic human inflammatory diseases. *Proc Natl Acad Sci U S A* 110: 3507–3512. 23401516 10.1073/pnas.1222878110 PMC3587220

[2] Mestas, J., and C.C.W. Hughes. 2004. Of Mice and Not Men: Differences between Mouse and Human Immunology. *The Journal of Immunology* 172: 2731–2738. 14978070 10.4049/jimmunol.172.5.2731

[3] Hanna, E., R. Abadi, and O. Abbas. 2016. Imiquimod in dermatology: An overview. *International Journal of Dermatology* 55: 831–844. 27387373 10.1111/ijd.13235

[4] van der Fits, L., S. Mourits, J.S.A. Voerman, et al. 2009. Imiquimod-Induced Psoriasis-Like Skin Inflammation in Mice Is Mediated via the IL-23/IL-17 Axis. *The Journal of Immunology* 182: 5836–5845. 19380832 10.4049/jimmunol.0802999

[5] Giacomassi, C., N. Buang, G.S. Ling, G. Crawford, H.T. Cook, D. Scott, F. Dazzi, J. Strid, and M. Botto. 2017. Complement C3 Exacerbates Imiquimod-Induced Skin Inflammation and Psoriasiform Dermatitis. *Journal of Investigative Dermatology* 137: 760–763. 27876407 10.1016/j.jid.2016.11.011 PMC5319416

[6] Flutter, B., and F.O. Nestle. 2013. TLRs to cytokines: Mechanistic insights from the imiquimod mouse model of psoriasis. *European Journal of Immunology* 43: 3138–3146. 24254490 10.1002/eji.201343801

[7] van der Kolk, T., S. Assil, R. Rijneveld, et al. 2018. Comprehensive, Multimodal Characterization of an Imiquimod-Induced Human Skin Inflammation Model for Drug Development. *Clinical and Translational Science* 11: 607–615. 29768709 10.1111/cts.12563 PMC6226121

[8] Assil, S., T.P. Buters, P.W. Hameeteman, et al. 2023. Oral prednisolone suppresses skin inflammation in a healthy volunteer imiquimod challenge model. *Frontiers in Immunology* 14: 1–11. 10.3389/fimmu.2023.1197650 PMC10400434 37545524

[9] R Core Team. 2021. R: A language and environment for statistical computing. R Foundation for Statistical Computing, Vienna, Austria. R version 4.3.1 (2023-06-16). https://www.R-project.org/.

[10] Van Der Maaten, L. 2014. Accelerating t-SNE using Tree-Based Algorithms. *The Journal of Machine Learning Research* 15: 3221–3245.

[11] Van Der Maaten, L., and G. Hinton. 2008. Visualizing data using t-SNE. *Journal of Machine Learning Research* 9: 2579–2625.

[12] Krijthe, J.H. 2015. Rtsne: T-distributed stochastic neighbor embedding using a barnes-hut implementation, R package version 0.16. https://github.com/jkrijthe/Rtsne.

[13] Love, M.I., W. Huber, and S. Anders. 2014. Moderated estimation of fold change and dispersion for RNA-seq data with DESeq2. *Genome Biology* 15: 1–21. 10.1186/s13059-014-0550-8 PMC4302049 25516281

[14] Zhu, A., J.G. Ibrahim, and M.I. Love. 2019. Heavy-tailed prior distributions for sequence count data: Removing the noise and preserving large differences. *Bioinformatics* 35: 2084–2092. 30395178 10.1093/bioinformatics/bty895 PMC6581436

[15] Wu, T., E. Hu, S. Xu, et al. 2021. clusterProfiler 4.0: A universal enrichment tool for interpreting omics data. *Innovation (Cambridge (Mass))* 2: 100141. 34557778 10.1016/j.xinn.2021.100141 PMC8454663

[16] Yu, G., L.-G. Wang, Y. Han, and Q.-Y. He. 2012. clusterProfiler: An R package for comparing biological themes among gene clusters. *OMICS: A Journal of Integrative Biology* 16: 284–287. 22455463 10.1089/omi.2011.0118 PMC3339379

[17] Subramanian, A., P. Tamayo, V.K. Mootha, et al. 2005. Gene set enrichment analysis: A knowledge-based approach for interpreting genome-wide expression profiles. *Proceedings of the National Academy of Sciences* 102: 15545–15550. 10.1073/pnas.0506580102 PMC1239896 16199517

[18] Liberzon, A., C. Birger, H. Thorvaldsdóttir, M. Ghandi, J.P. Mesirov, and P. Tamayo. 2015. The molecular signatures database hallmark gene set collection. *Cell Systems* 1: 417–425. 26771021 10.1016/j.cels.2015.12.004 PMC4707969

[19] Hänzelmann, S., R. Castelo, and J. Guinney. 2013. GSVA: Gene set variation analysis for microarray and RNA-Seq data. *BMC Bioinformatics*. 10.1186/1471-2105-14-7. 23323831 10.1186/1471-2105-14-7 PMC3618321

[20] Kolde, R., and M.R. Kolde. 2015. Package ‘pheatmap.’ *R package* 1: 790.

[21] Yu, G. 2024. Enrichplot: Visualization of functional enrichment result. 10.18129/B9.bioc.enrichplot, R package version 1.20.3. https://bioconductor.org/packages/release/bioc/html/enrichplot.html.

[22] Newman, A.M., C.B. Steen, C.L. Liu, et al. 2019. Determining cell type abundance and expression from bulk tissues with digital cytometry. *Nature Biotechnology* 37: 773–782. 31061481 10.1038/s41587-019-0114-2 PMC6610714

[23] Prens, L.M., C.B. Ardon, K.R. van Straalen, H.H. van der Zee, M.A.J. Seelen, J.D. Laman, E.P. Prens, B. Horváth, and J. Damman. 2021. No Evident Systemic Terminal Complement Pathway Activation in Hidradenitis Suppurativa. *Journal of Investigative Dermatology* 141: 2966-2969.e1. 34252397 10.1016/j.jid.2021.03.037

[24] Gorden, K.B., K.S. Gorski, S.J. Gibson, R.M. Kedl, W.C. Kieper, X. Qiu, M.A. Tomai, S.S. Alkan, and J.P. Vasilakos. 2005. Synthetic TLR Agonists Reveal Functional Differences between Human TLR7 and TLR8. *The Journal of Immunology* 174: 1259–1268. 15661881 10.4049/jimmunol.174.3.1259

[25] Buters, T.P., P.W. Hameeteman, I.M.E. Jansen, et al. 2022. Clinical, Cellular, and Molecular Effects of Corticosteroids on the Response to Intradermal Lipopolysaccharide Administration in Healthy Volunteers. *Clinical Pharmacology and Therapeutics* 111: 964–971. 34935141 10.1002/cpt.2516 PMC9305467

[26] Buters, T.P., P.W. Hameeteman, I.M.E. Jansen, et al. 2022. Intradermal lipopolysaccharide challenge as an acute in vivo inflammatory model in healthy volunteers. *British Journal of Clinical Pharmacology* 88: 680–690. 34293819 10.1111/bcp.14999 PMC9290695

[27] Dillingh, M.R., E.P. Van Poelgeest, K.E. Malone, E.M. Kemper, E.S.G. Stroes, M. Moerland, and J. Burggraaf. 2014. Characterization of inflammation and immune cell modulation induced by low-dose LPS administration to healthy volunteers. *Journal of Inflammation (United Kingdom)* 11: 1–9.

[28] van Poelgeest, E.P., M.R. Dillingh, M. de Kam, K.E. Malone, M. Kemper, E.S.G. Stroes, J. Burggraaf, and M. Moerland. 2018. Characterization of immune cell, endothelial, and renal responses upon experimental human endotoxemia. *Journal of Pharmacological and Toxicological Methods* 89: 39–46. 29056520 10.1016/j.vascn.2017.10.004

[29] Majoros, A., E. Platanitis, E. Kernbauer-Hölzl, F. Rosebrock, M. Müller, and T. Decker. 2017. Canonical and non-canonical aspects of JAK-STAT signaling: Lessons from interferons for cytokine responses. *Frontiers in Immunology*. 10.3389/fimmu.2017.00029. 28184222 10.3389/fimmu.2017.00029 PMC5266721

[30] Mahjoor, M., G. Mahmoudvand, S. Farokhi, A. Shadab, M. Kashfi, and H. Afkhami. 2023. Double-edged sword of JAK/STAT signaling pathway in viral infections: Novel insights into virotherapy. *Cell Communication and Signaling* 21: 1–17. 37784164 10.1186/s12964-023-01240-y PMC10544547

[31] Nickles, M.A., K. Huang, Y.S. Chang, M.M. Tsoukas, N.J. Sweiss, D.L. Perkins, and P.W. Finn. 2020. Gene Co-expression Networks Identifies Common Hub Genes Between Cutaneous Sarcoidosis and Discoid Lupus Erythematosus. *Front Med (Lausanne)* 7: 1–8. 33324666 10.3389/fmed.2020.606461 PMC7724034

[32] Solé, C., M. Gimenez-Barcons, B. Ferrer, J. Ordi-Ros, and J. Cortés-Hernández. 2016. Microarray study reveals a transforming growth factor-β-dependent mechanism of fibrosis in discoid lupus erythematosus. *British Journal of Dermatology* 175: 302–313. 26972571 10.1111/bjd.14539

[33] Blomberg, S., L. Rönnblom, M.L. Eloranta, B. Cederblad, G.V. Alm, K. Nordlind, and K. Nordlind. 2001. Presence of cutaneous interferon-α producing cells in patients with systemic lupus erythematosus. *Lupus* 10: 484–490. 11480846 10.1191/096120301678416042

[34] Wongpiyabovorn, J., K. Ruchusatsawat, Y. Onganantapong, W. Sintupak, and N. Hirankarn. 2011. Interferon Alpha mRNA level and subtypes in lesion and non-lesion from discoid lupus erythematosus patients without systemic lupus erythematosus. *Asian Biomedicine* 5: 643–647.

[35] Kittaka, H., and M. Tominaga. 2017. The molecular and cellular mechanisms of itch and the involvement of TRP channels in the peripheral sensory nervous system and skin. *Allergology International* 66: 22–30. 28012781 10.1016/j.alit.2016.10.003

[36] Bhagchandani, S., J.A. Johnson, and D.J. Irvine. 2021. Evolution of Toll-like receptor 7/8 agonist therapeutics and their delivery approaches: From antiviral formulations to vaccine adjuvants. *Advanced Drug Delivery Reviews*. 10.1016/j.addr.2021.05.013. 34058283 10.1016/j.addr.2021.05.013 PMC9003539

[37] Hayashi, F., T.K. Means, and A.D. Luster. 2003. Toll-like receptors stimulate human neutrophil function. *Blood* 102: 2660–2669. 12829592 10.1182/blood-2003-04-1078

[38] Trinchieri, G., and A. Sher. 2007. Cooperation of Toll-like receptor signals in innate immune defence. *Nature Reviews Immunology* 7: 179–190. 17318230 10.1038/nri2038

