# Supplementary material for: Extending the IMQ Model: Deep Characterization of the Human TLR7 Response for Early Drug Development
Source: Inflammation. 2024 Aug 26;48(3):1366–77. doi: 10.1007/s10753-024-02127-x (PMC12234578; doi:10.1007/s10753-024-02127-x)
Supplement: Supplementary file 1 — Supplementary file1 (DOCX 2519 KB) [file 10753_2024_2127_MOESM1_ESM.docx]

**Supplementary material**

**Table S1**. Overview of samples analysed using RNA sequencing.

| **SampleID** | **Sample** | **Subjectnr** | **Timepoint** | **Group** | **Responder  (molecular)** |
| --- | --- | --- | --- | --- | --- |
| 105669-001-004 | S6_Untreated | 6 | Untreated | Untreated |  |
| 105669-001-009 | S1_Untreated | 1 | Untreated | Untreated |  |
| 105669-001-014 | S2_Untreated | 2 | Untreated | Untreated |  |
| 105669-001-019 | S3_Untreated | 3 | Untreated | Untreated |  |
| 105669-001-024 | S4_Untreated | 4 | Untreated | Untreated |  |
| 105669-001-029 | S5_Untreated | 5 | Untreated | Untreated |  |
| 105669-001-034 | S7_Untreated | 7 | Untreated | Untreated |  |
| 105669-001-049 | S10_Untreated | 10 | Untreated | Untreated |  |
| 105669-001-001 | S6_IMQ48h* | 6 | IMQ48h | Short exposure (48h + 72h) | no |
| 105669-001-006 | S1_IMQ48h* | 1 | IMQ48h | Short exposure (48h + 72h) | no |
| 105669-001-011 | S2_IMQ48h | 2 | IMQ48h | Short exposure (48h + 72h) | yes |
| 105669-001-016 | S3_IMQ48h | 3 | IMQ48h | Short exposure (48h + 72h) | yes |
| 105669-001-021 | S4_IMQ48h | 4 | IMQ48h | Short exposure (48h + 72h) | yes |
| 105669-001-026 | S5_IMQ48h | 5 | IMQ48h | Short exposure (48h + 72h) | yes |
| 105669-001-031 | S7_IMQ48h | 7 | IMQ48h | Short exposure (48h + 72h) | yes |
| 105669-001-036 | S8_IMQ48h | 8 | IMQ48h | Short exposure (48h + 72h) | yes |
| 105669-001-041 | S9_IMQ48h | 9 | IMQ48h | Short exposure (48h + 72h) | yes |
| 105669-001-046 | S10_IMQ48h* | 10 | IMQ48h | Short exposure (48h + 72h) | no |
| 105669-001-002 | S6_IMQ72h* | 6 | IMQ72h | Short exposure (48h + 72h) | no |
| 105669-001-007 | S1_IMQ72h* | 1 | IMQ72h | Short exposure (48h + 72h) | no |
| 105669-001-017 | S3_IMQ72h* | 3 | IMQ72h | Short exposure (48h + 72h) | no |
| 105669-001-020 | S2_IMQ72h* | 2 | IMQ72h | Short exposure (48h + 72h) | no |
| 105669-001-022 | S4_IMQ72h* | 4 | IMQ72h | Short exposure (48h + 72h) | no |
| 105669-001-027 | S5_IMQ72h | 5 | IMQ72h | Short exposure (48h + 72h) | yes |
| 105669-001-032 | S7_IMQ72h | 7 | IMQ72h | Short exposure (48h + 72h) | yes |
| 105669-001-037 | S8_IMQ72h | 8 | IMQ72h | Short exposure (48h + 72h) | yes |
| 105669-001-047 | S10_IMQ72h | 10 | IMQ72h | Short exposure (48h + 72h) | yes |
| 105669-001-003 | S6_IMQ120h* | 6 | IMQ120h | Long exposure (120h + 168h) | no |
| 105669-001-008 | S1_IMQ120h | 1 | IMQ120h | Long exposure (120h + 168h) | yes |
| 105669-001-013 | S2_IMQ120h* | 2 | IMQ120h | Long exposure (120h + 168h) | no |
| 105669-001-018 | S3_IMQ120h* | 3 | IMQ120h | Long exposure (120h + 168h) | no |
| 105669-001-023 | S4_IMQ120h | 4 | IMQ120h | Long exposure (120h + 168h) | yes |
| 105669-001-028 | S5_IMQ120h | 5 | IMQ120h | Long exposure (120h + 168h) | yes |
| 105669-001-033 | S7_IMQ120h* | 7 | IMQ120h | Long exposure (120h + 168h) | no |
| 105669-001-043 | S9_IMQ120h | 9 | IMQ120h | Long exposure (120h + 168h) | yes |
| 105669-001-048 | S10_IMQ120h | 10 | IMQ120h | Long exposure (120h + 168h) | yes |
| 105669-001-005 | S6_IMQ168h* | 6 | IMQ168h | Long exposure (120h + 168h) | no |
| 105669-001-010 | S1_IMQ168h | 1 | IMQ168h | Long exposure (120h + 168h) | yes |
| 105669-001-012 | S3_IMQ168h* | 3 | IMQ168h | Long exposure (120h + 168h) | no |
| 105669-001-015 | S2_IMQ168h | 2 | IMQ168h | Long exposure (120h + 168h) | yes |
| 105669-001-025 | S4_IMQ168h | 4 | IMQ168h | Long exposure (120h + 168h) | yes |
| 105669-001-030 | S5_IMQ168h | 5 | IMQ168h | Long exposure (120h + 168h) | yes |
| 105669-001-035 | S7_IMQ168h* | 7 | IMQ168h | Long exposure (120h + 168h) | no |
| 105669-001-040 | S8_IMQ168h | 8 | IMQ168h | Long exposure (120h + 168h) | yes |
| 105669-001-045 | S9_IMQ168h | 9 | IMQ168h | Long exposure (120h + 168h) | yes |

***Table S2****. Overview of subject demographics*

| **All subjects** | |
| --- | --- |
| **Age (years)** | |
| N | 10 |
| Mean (SD) | 25.6 (6.7) |
| Median | 24 |
| Min, Max | 18, 37 |
| **Height (cm)** | |
| N | 10 |
| Mean (SD) | 176.57 (7.48) |
| Median | 176.0 |
| Min, Max | 164.9, 192.0 |
| **Weight (kg)** | |
| N | 10 |
| Mean (SD) | 77.290 (13.677) |
| Median | 77.68 |
| Min, Max | 57.40, 95.50 |
| **BMI (kg/m2)** | |
| N | 10 |
| Mean (SD) | 24.68 (3.33) |
| Median | 24.7 |
| Min, Max | 20.1, 30.0 |
| **Sex** | |
| Female | 7 (70.0%) |
| Male | 3 (30.0%) |

*
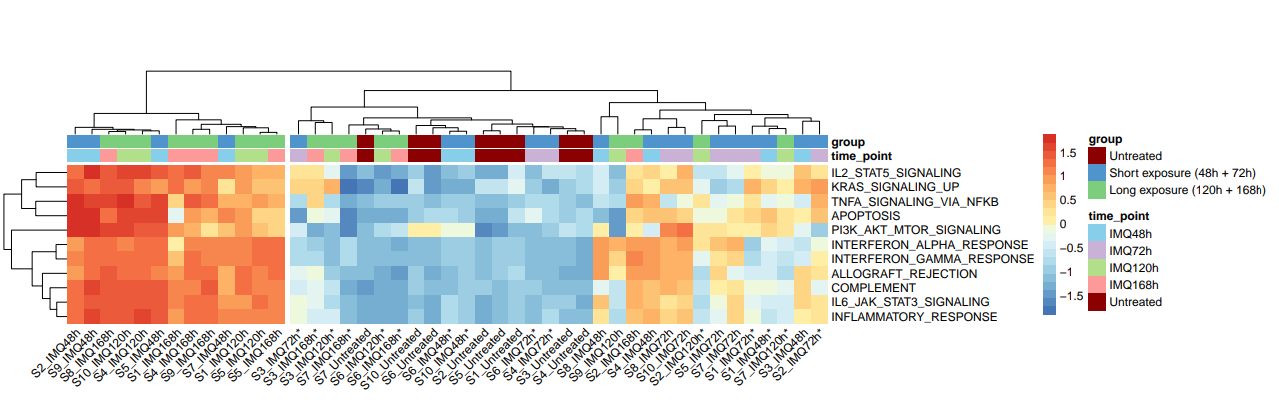
*

***Figure S1****.Heatmap of GSVA single-sample pathway enrichment scores on 11 representative MSigDB Hallmark gene sets, scaled by row and shown across the full data set (n=45 samples).*


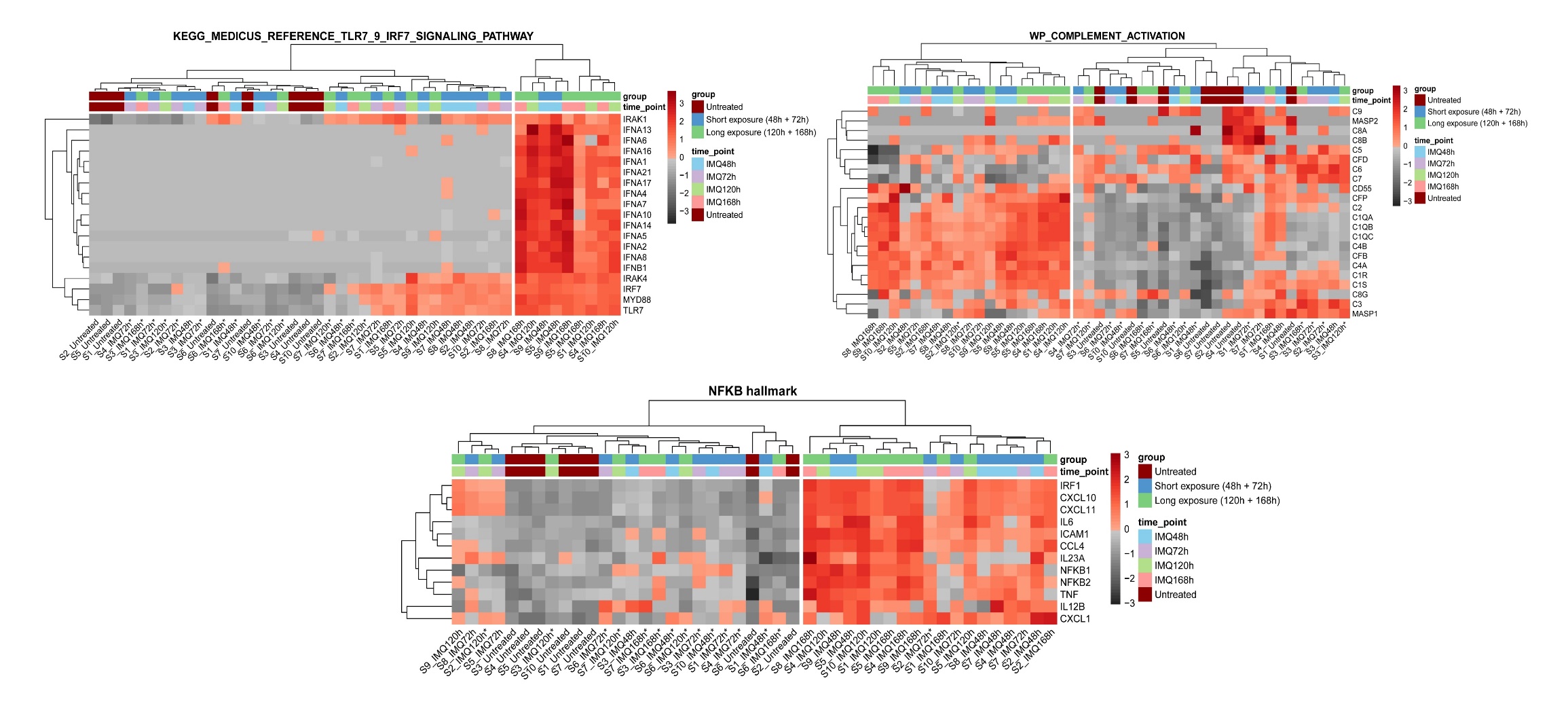


**A**

**B**

**C**

**Figure S2**. Key pathways involved in the IMQ response. Gene expression for members of the **A** TLR7/9 signalling pathway (KEGG Medicus), **B** complement activation pathway (WP; WikiPathways), **C** a subset of the TNF signalling via NF-kB pathway (Hallmark gene sets); scaled by row across the full dataset (n=45 samples).

**Figure S3.** Staining of biopsies for **A** acanthosis **B** lymphocytic exocytosis **C** NF-κB **D** B cells **E** neutrophils **F** Langerhans cells.


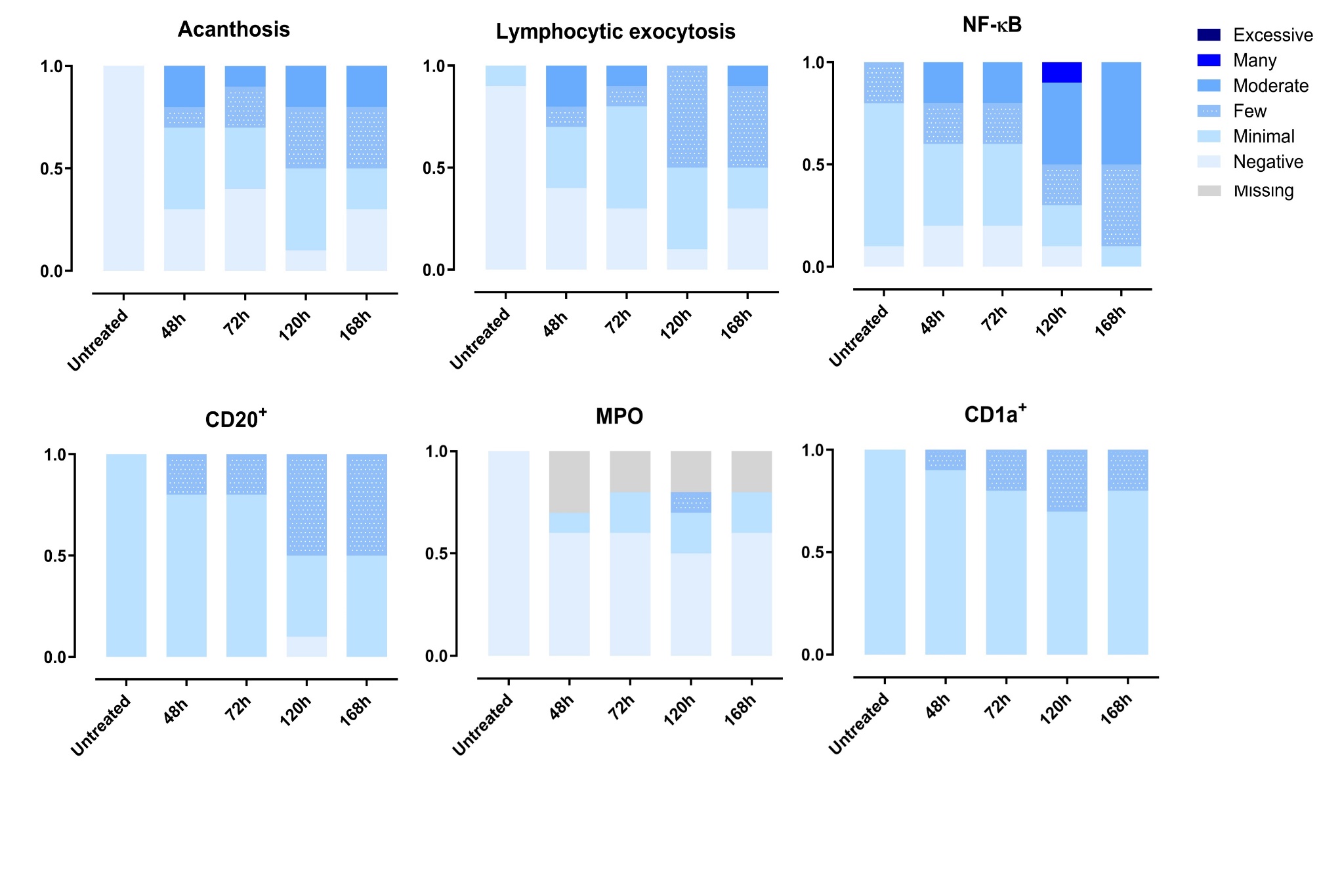


**A**

**B**

**C**

**D**

**E**

**F**


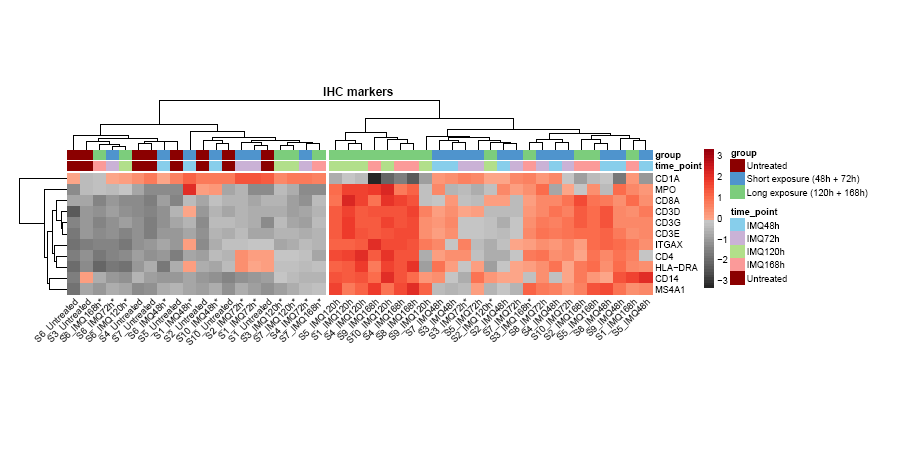


**Figure S4**. Expression of transcripts corresponding to the markers used for IHC, scaled by row and shown across the full data set (n=45 samples).


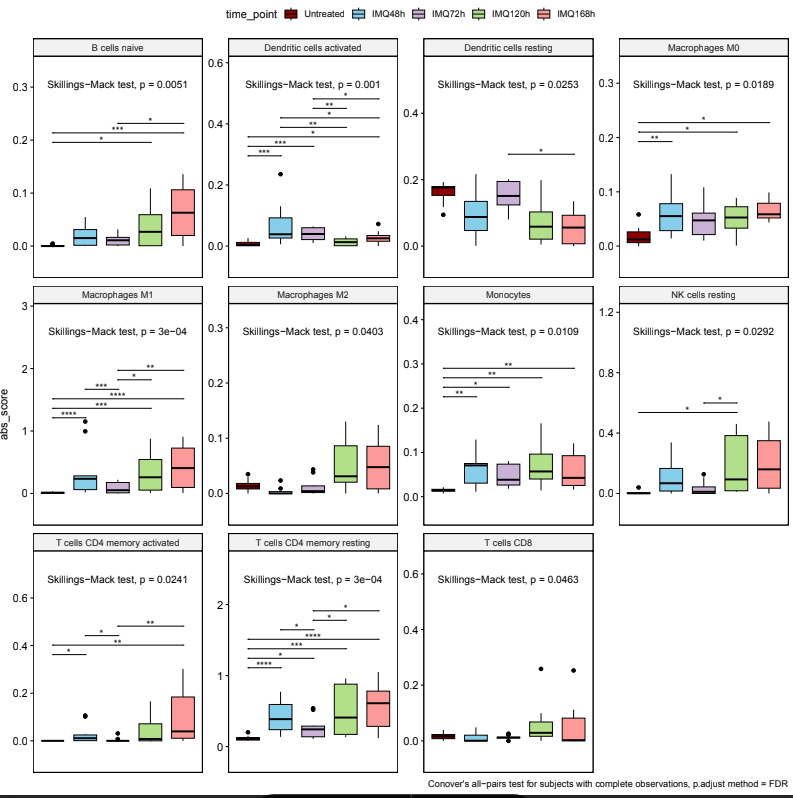


**Figure S5.** Overview of cell types profiled using CIBERSORTx per time point. Boxplots depicting absolute scores for 11 cell types with statistically significant changes across time points (Skillings-Mack test). The Conover’s all-pairs test with FDR correction was applied as the post hoc test using data from subjects with complete observations. Three subjects were excluded from the analysis due to incomplete observations.
